# Complete Genome Sequence of *Geobacillus* sp. Strain GHH01, a Thermophilic Lipase-Secreting Bacterium

**DOI:** 10.1128/genomeA.00092-13

**Published:** 2013-04-25

**Authors:** Sandra Wiegand, Ulrich Rabausch, Jennifer Chow, Rolf Daniel, Wolfgang R. Streit, Heiko Liesegang

**Affiliations:** Department of Genomic and Applied Microbiology, Göttingen Genomics Laboratory, Institut für Mikrobiologie und Genetik, Norddeutsches Zentrum für Mikrobielle Genomforschung, Georg-August-Universität, Göttingen, Germanya;; Abteilung für Mikrobiologie und Biotechnologie, Biozentrum Klein Flottbek, Universität Hamburg, Hamburg, Germanyb

## Abstract

*Geobacillus* sp. strain GHH01 was isolated during a screening for producers of extracellular thermostable lipases. The completely sequenced and annotated 3.6-Mb genome encodes 3,478 proteins. The strain is genetically equipped to utilize a broad range of different substrates and might develop natural competence.

## GENOME ANNOUNCEMENT

The genus *Geobacillus* contains thermophilic strains, which produce a variety of thermostable hydrolytic extracellular enzymes, such as proteases, amylases, and lipases. These features are interesting for future production platforms used in industrial applications (1).

Here, we present the complete genome sequence of *Geobacillus* sp. strain GHH01, a thermophilic lipase producer. The strain was isolated from an enrichment culture originally sampled at Botanischer Garten, University of Hamburg, Germany, and was cultivated at 60°C with 1.5% native olive oil as the sole carbon source. Recombinant expression in *Escherichia coli* revealed that the *Geobacillus* sp. GHH01 lipase (locus tag GHH_c20570) is highly active but only moderately thermostable.

The genome sequence of *Geobacillus* sp. GHH01 was determined by a combined approach of 454 GS-FLX Titanium XL paired-end sequencing (454 Life Sciences, Branford, CT) and Genome Analyzer II single-read sequencing (TruSeq Chemistry, Illumina, San Diego, CA), resulting in average coverages of 10.91-fold and 33.03-fold, respectively. The assembly employing the MIRA v3.4.1.1 software (2) yielded 84 contigs >3 kbp. Gap closure and quality improvement were performed by PCR-based techniques and subsequent Sanger sequencing (ABI 3730xl, Life Technologies, Carlsbad, CA). Initial gene prediction was performed with IMG/ER (3), followed by manual curation based on comparisons to the Swiss-Prot, TrEMBL (4), and InterPro (5) databases. For the identification of rRNA and tRNA genes, RNAmmer v1.2 and tRNAscan-SE v1.4 (6, 7) were used, respectively.

The complete genome consists of a 3,582,992-bp chromosome with a G+C content of 52.3%. In total, 3,597 genes were identified, including 10 rRNA gene clusters and 88 tRNA genes. The annotation resulted in 2,724 protein-encoding genes with assigned functions.

16S rRNA gene phylogenetic analysis confirmed the affiliation of *Geobacillus* sp. GHH01 to the genus *Geobacillus*, whereas an assignment to a described species was not possible. We determined average nucleotide identities (8) of approximately 96% between the *Geobacillus* sp. GHH01 genome and the genomes of *Geobacillus kaustophilus* HTA426 (9) and *Geobacillus thermoleovorans* CCB_US3_UF5 (10). The recently mentioned (10) high synteny between *G. thermoleovorans* CCB_US3_UF5 and *G. kaustophilus* HTA426 (97.94%) calls into question their assignment to distinct species. Hence, a sequence similarity-based assignment of *Geobacillus* sp. GHH01 to a distinct species could not be employed.

*Geobacillus* sp. GHH01 is predicted to secrete 139 enzymes by the Sec-dependent pathway (11, 12), including the identified lipase, diverse peptidases, proteinases, an amylopullanase (GHH_c32620), an alpha-amylase (GHH_c32630), and an alkaline phosphatase (GHH_c27900). Several substrate-binding proteins of ABC transporters indicate the potential for utilization of a broad range of substrates. The ability to take up extracellular DNA is a crucial mechanism for strain development. Eighteen out of 25 main competence-related structural genes identified for *Bacillus subtilis* (13) were detected, featuring a possible mechanism of DNA uptake.

Genome comparisons revealed seven distinct GHH01-specific genomic islands (14). Furthermore, 123 putative transposases, five clustered regularly interspaced short palindromic repeat (CRISPR) regions, and nine CRISPR-associated genes of subtype III-B (15) could be detected.

### Nucleotide sequence accession number. 

The genome sequence of *Geobacillus* sp. GHH01 has been deposited in GenBank under accession no. CP004008. The strain is available upon request at the Bacillus Genetic Stock Center (BGSC). 
